# Polymorphisms in Genes Affecting Interferon-γ Production and Th1 T Cell Differentiation Are Associated With Progression to Chagas Disease Cardiomyopathy

**DOI:** 10.3389/fimmu.2020.01386

**Published:** 2020-07-07

**Authors:** Amanda Farage Frade-Barros, Barbara Maria Ianni, Sandrine Cabantous, Cristina Wide Pissetti, Bruno Saba, Hui Tzu Lin-Wang, Paula Buck, José Antonio Marin-Neto, André Schmidt, Fabrício Dias, Mario Hiroyuki Hirata, Marcelo Sampaio, Abílio Fragata, Alexandre Costa Pereira, Eduardo Donadi, Virmondes Rodrigues, Jorge Kalil, Christophe Chevillard, Edecio Cunha-Neto

**Affiliations:** ^1^Heart Institute (InCor), University of São Paulo School of Medicine (FMUSP), São Paulo, Brazil; ^2^Institute for Investigation in Immunology (iii), INCT, São Paulo, Brazil; ^3^Aix-Marseille Université, INSERM, GIMP UMR_S906, Marseille, France; ^4^Division of Clinical Immunology and Allergy, University of São Paulo School of Medicine, São Paulo, Brazil; ^5^Bioengineering Program, Instituto Tecnológico, Universidade Brasil, São Paulo, Brazil; ^6^Laboratory of Immunology, Universidade Federal Do Triângulo Mineiro (UFTM), Uberaba, Brazil; ^7^Laboratório de Investigação Molecular em Cardiologia, Instituto de Cardiologia Dante Pazzanese (IDPC), São Paulo, Brazil; ^8^School of Medicine of Ribeirão Preto (FMRP), University of São Paulo, Ribeirão Preto, Brazil; ^9^Department of Clinical and Toxicological Analyses, Faculty of Pharmaceutical Sciences, University of São Paulo (USP), São Paulo, Brazil; ^10^Aix Marseille Université, INSERM, TAGC Theories and Approaches of Genomic Complexity, UMR_1090, Marseille, France

**Keywords:** Chagas disease, cardiomyopathy, susceptibility, IL12, IL 10, IFN, IL4

## Abstract

**Background:** Chagas disease, caused by the protozoan *Trypanosoma cruzi*, is endemic in Latin America. Thirty percent of infected individuals develop chronic Chagas cardiomyopathy (CCC), an inflammatory dilated cardiomyopathy that is the most important clinical consequence of *T. cruzi* infection, while the others remain asymptomatic (ASY). IFN-γ and IFN-γ-producing Th1-type T cells are increased in peripheral blood and CCC myocardium as compared to ASY patients, while the Th1-antagonizing cytokine IL-10 is more expressed in ASY patients. Importantly IFN-γ-producing Th1-type T cells are the most frequent cytokine-producing T cell subset in CCC myocardium, while expression of Th1-antagonizing cytokines IL-10 and IL-4 is unaltered. The control of IFN-γ production by Th1-type T cells may be a key event for progression toward CCC. A genetic component to disease progression was suggested by the familial aggregation of cases and the association of gene polymorphisms with CCC development. We here investigate the role of gene polymorphisms (SNPs) in several genes involved in the control of IFN-γ production and Th1 T cell differentiation in CCC development.

**Methods:** We studied a Brazilian population including 315 CCC cases and 118 ASY subjects. We assessed 35 Tag SNPs designed to represent all the genetic information contained in the *IL12B, IL10, IFNG*, and *IL4* genes.

**Results:** We found 2 *IL12* SNPs (rs2546893, rs919766) and a trend of association for a *IL10* SNP (rs3024496) to be significantly associated with the ASY group. these associations were confirmed by multivariate analysis and allele tests. The rs919766C, 12rs2546893G, and rs3024496C alleles were associated to an increase risk to CCC development.

**Conclusions:** Our data show that novel polymorphisms affecting *IL12B* and *IL10*, but not *IFNG* or *IL4* genes play a role in genetic susceptibility to CCC development. This might indicate that the increased Th1 differentiation and IFN-γ production associated with CCC is genetically controlled.

## Introduction

Chagas disease (American trypanosomiasis) is caused by the protozoan *Trypanosoma cruzi* (*T. cruzi*) and mainly transmitted by the reduviid arthropod vector. It is endemic in Latin America, where an estimated 8 million people are infected (https://www.cdc.gov/parasites/chagas/index.html). Imported disease is increasingly recognized as an emerging problem in the USA and Europe due to immigration from Latin America (https://www.who.int/chagas/disease/en/). Decades after acute infection, 20–30% of the patients develop chronic Chagas disease cardiomyopathy (CCC), where heart tissue inflammation and remodeling, fibrosis, electrical and structural abnormalities often lead to life-threatening heart failure and arrhythmia (1). Five to 10% of infected individuals develop digestive disease, with denervation and dilation of the digestive tract. The remaining two-thirds of infected individuals remain asymptomatic (ASY) and free from symptomatic disease for life (2). Heart failure due to CCC has a worse prognosis, with 50% shorter survival when compared to like ischemic, idiopathic and hypertensive cardiomyopathies (3, 4). The pathogenic determinants of differential disease progression have not yet been completely elucidated.

The finding that 30% of Chagas disease patients develop CCC suggested the participation of genetics in differential disease progression. In addition, familial aggregation of CCC cases has been described (5), and a significant number of genetic polymorphisms has been associated with CCC development. A recent literature search found ca. 150 case-control genetic association studies addressing polymorphisms in 76 genes, which disclosed 62 SNPs from 44 genes to be associated with CCC, mostly related to immunology and inflammation. From those, a much smaller number of SNPs were associated with CCC severity (ventricular dysfunction) (6–8).

Many of these SNPs are related to pathogen resistance and disease tolerance genes—those related to direct control of parasitism or to reduction of tissue damage, respectively (7). Chagas disease is associated with increased production of inflammatory cytokines; IFN-γ is a major pathogen resistance gene and plays a major role in the disease; *T. cruzi* infection of mice genetically deficient of IFNG leads to uncontrolled parasitism and 100% mortality (9, 10). While it is a key player in pathogen protection, excessive levels lead to increased inflammation and tissue damage during the acute phase as well as CCC (7). During acute infection, *T. cruzi* pathogen-associated molecular patterns (PAMPs) trigger innate immunity (3, 11, 12) leading to the release of proinflammatory cytokines and chemokines, including IL-12 and IL-18, the major drivers of differentiation of IFN-γ-producing *T. cruzi*–specific Th1 T cells which migrate to sites of *T. cruzi*-induced inflammation (13, 14). IFNγ is then produced at high levels, inducing production of microbicidal and potentially tissue-damaging reactive oxygen and nitrogen species that kill intracellular *T. cruzi*. This leads to the control—but not complete elimination—of parasitism, establishing a low-grade chronic persistent infection by *T. cruzi*. On the other hand, cytokines that are able to modulate excessive IFN-γ production/Th1 T cell differentiation can modulate tissue damage in the acute phase of infection. Mice genetically deficient in disease tolerance genes IL10 (15), IL4 (16) and IL27b/Ebi3 (17), factors known to limit Th1 T cell differentiation and IFNγ production (17–20) display increased inflammation and may die early. Animals who keep a balance between inflammatory Th1 and anti-inflammatory cytokines may survive and progress to the chronic phase. As a result of persistent infection, both CCC and ASY chronic Chagas disease patients produce higher levels of IFNγ than seronegative controls (21, 22), but those who develop Chagas cardiomyopathy display a particularly strong Th1-type immune response with increased numbers of IFN-γ-producing Th1 cells in peripheral blood mononuclear cells (PBMC) (19, 20, 22, 23) and decreased IFNγ -modulating factors IL-10, IL-27b/Ebi3, and Treg cells (17–20). Accordingly, CCC patients displayed increased plasma levels of IFNγ while levels of IL-10 are reduced when compared to ASY individuals (24–26). These data suggest that the cytokine balance can modulate progression to CCC development. Indeed, CCC hearts display a prominent IFNγ -producing T-cell dependent myocarditis. IFNγ is the most highly expressed cytokine in CCC myocardium (27), and mononuclear cells producing this cytokine are abundant in CCC heart (12, 14, 22). mRNA expression IL-10 and other Th1-antagonizing cytokines and cell types (Treg and Th2) was low or undetectable in CCC heart tissue. This indicates that the Th1 infiltrate in CCC myocardium is essentially unopposed by regulatory cells or cytokines, and subject to little regulation (27). The failure to regulate Th1 responses and IFNγ production may thus be one key immune defect of patients who progress to CCC. This creates a paradox, in that IFNγ is essential for control of parasitism in acute and chronic Chagas disease, but at the same time causes tissue damage (7).

Cytokine gene polymorphisms may contribute to the control of Th1 T cell differentiation/IFN-γ production. Previous studies have assessed the association of SNPs in cytokines important for regulation of IFN-γ responses with CCC. For *IFNG*, no SNP was found to be associated with CCC when compared to ASY after comparison for multiple testing (28). Likewise, *IL4* SNP case-control studies between CCC and ASY failed to show associations, but may have been underpowered, with only110 and 260 total Chagasic patients, respectively (29, 30). A promoter polymorphism in *IL12B* +1188 (rs3212227) was found to be associated with CCC in an Colombian population (31). The SNP IL10-1082 (rs1800896) was shown to be functional (32) and was associated to CCC in a Brazilian population comparing ASY individuals vs. CCC (33). On a Brazilian population, the frequency of the polymorphic rs1800896A allele (associated with lower expression of IL-10) was higher in the asymptomatic group than in the cardiac group. On the other hand, Florez et al. compared CCC (*n* = 130) to ASY (*n* = 130) cases in a Colombian population. Florez et al. failed to show associations in the genotypes and allele frequencies neither in *IL10* SNPs *IL10*-1082 (rs1800896) nor in the additional SNPs *IL10*-819 (rs1800871) and *IL10*-592 (rs1800872). It is possible that this lack of association in our study is was due to a small sample size or to epistasis between IL-10 and MHC.

Since the previous studies had some limitations, i.e., focused on selected polymorphisms, failed to be replicated in other populations, or lacked appropriate statistical power, we here comprehensively assessed the role of polymorphisms in these genes on CCC progression. For that matter we here performed a thorough genetic study focusing on 35 polymorphisms of *IL4, IL12B, IL10*, and *IFNG* using the Tag SNP approach, which represent all the genetic information contained in the mentioned genes, in a larger cohort (*n* = 433) of Brazilian Chagas disease patients, including CCC patients with or without ventricular dysfunction as well as asymptomatic patients. With the tag SNP approach, we could both replicate the study of previously investigated SNPs as well as novel polymorphisms. This way, we could perform a more sensitive assessment of the contribution of genetic variants in prognosis to CCC either confirming or finding additional associated SNPs in the mentioned genes. In addition, our experimental design allowed the study of possible interactions between polymorphisms in different cytokines.

## Methods

### Ethical Standard

Written informed consent was obtained from all the patients, in accordance with the guidelines of the various internal review boards of all the involved institutions. The protocol was also approved by the INSERM Internal Review Board and the Brazilian National Ethics in Research Commission (CONEP). All the patients enrolled in this study were over 21 years old. Investigations were conformed to the principles outlined in the declaration of Helsinki.

### Diagnostic Criteria

The diagnostic criteria for Chagas disease included the detection of antibodies against *T. cruzi* in at least two of three independent serological tests (EIA [Hemobio Chagas; Embrabio São Paulo], indirect immunofluorescence assays [IFA-immunocruzi; Biolab Merieux], and indirect hemagglutination tests [Biolab Merieux]) (34). All Chagas disease patients underwent standard electrocardiography and echocardiography. Echocardiography was performed at the hospital, with a Sequoia model 512 echocardiograph with a broad-band transducer. Left ventricular dimensions and regional and global function, including the recording of left ventricular ejection fraction (LVEF), were evaluated with a two-dimensional, M-mode approach, in accordance with the recommendations of the American Society of Echocardiography. ASY subjects had no electrocardiography and echocardiography changes. CCC patients presented typical conduction abnormalities (right bundle branch block and/or left anterior division hemiblock) (35). CCC patients with significant left ventricular systolic dysfunction (LVEF <40%) were classified as having severe CCC, whereas those with no significant ventricular dysfunction (LVEF ≥ 40%) were classified as having moderate CCC. We selected 40% as arbitrary cutoff value that has been previously used to define significant ventricular dysfunction by our group and others (36–38).

### Study Population for Polymorphism Analysis

The CCC and ASY patients were born and raised in rural areas of Sao Paulo, Minas Gerais and Bahia states and enrolled in one of the study centers (Incor, FMUSP, FMRP, UFTM, IDPC). Patients with digestive forms were excluded of this study. Patients were classified as ASY (*n* = 118) or as having CCC (*n* = 315). ASY individuals were used as the control subjects for this study because they were from the same areas of endemicity as the patients with CCC, had encountered the parasite and had tested seropositive for T. cruzi infection, but the infection had not progressed to CCC. Here, the Case/Control ratio, which is 0.375, illustrate the difficulty to enroll real ASY subjects. For our association study, if we are fixing the significant level to 0.05, the disease prevalence to 30%, the disease allele Frequency to 0.2 and the genotype relative risk to 1.5, the expected power of our association study reaches 0.895.

Of 315 patients with CCC, 106 (42 men [39.6%] and 64 women [60.4%]) showed no significant ventricular dysfunction (LVEF ≥ 40%) and were thus classified as having moderate CCC, whereas 199 (144 men [72.4%] and 55 women [27.6%]) had severe ventricular dysfunction (LVEF <40%) and were classified as having severe CCC. Data for left ventricular ejection fraction were missing for 10 patients with CCC. So, when we compared moderate patients to severe patients, these 10 individuals were excluded from the analysis. Regarding progression of the ASY cases to CCC, the yearly progression rate –regardless of age group- is ca. 1–2%/year. The average age of Subjects with asymptomatic form was above 55 years. Taking into account that they were all born in endemic areas before vector transmission was interrupted, it is likely that in most if not all cases vector-borne infection occurred in early childhood. The odds that a significant number of such mature patients convert to CCC, and that this thwarts our statistical calculation is rather low; however, this is a pitfall of all cross sectional studies on diseases that display progression.

### Blood Samples and DNA Preparation

For each subject, 5–15 ml of blood were collected in EDTA tubes. Genomic DNA was isolated on a silica-membrane according to the manufacturer's protocol (QIAamp DNA Blood Max Kit, Qiagen, Hilden, Germany).

### SNP Selection

Tag single nucleotide polymorphisms (SNPs) were selected on the basis on Ensembl Release GRCh38 for the Caucasian and Yoruba reference populations. Tag SNPs were selected within a region extending 5 kb on either side of the candidate gene. The minor allele frequency (MAF) cut off value was arbitrarily set at 20% (so the markers characterized by a MAF <20% were excluded from the analysis by lack of power). In each reference population, the markers with MAF > 20% are included in different blocks of correlation (based on the *r*^2^ values). For each correlation block in the gene, one marker was selected and considered as a Tag SNP. Indeed, markers located in the same block of correlation yielded the same genetic information in association studies. Tag SNPs characterized by a MAF over 20% on at least one reference population were selected. These Tag SNPs were thus defined to gather all the genetic information from each candidate gene. we selected a total of 35 Tag SNPs: sixteen tag SNPs for *IL12B*, six tag SNPs for *IL10*, six for *IFNG* and seven tag SNPs for *IL4*.

### SNP Genotyping

Most of the genotyping was done with the Golden Gate genotyping assay (Illumina, San Diego, USA). In some cases, genotyping assays were performed with the *Taq*Man system (Applied Biosystems, Foster City, USA) according to the manufacturer's instructions. Genotyes are available on request.

### Statistical Analysis

SPSS Statistics software v. 17.0 (IBM, Armonk, USA) was used for statistical analyses. We performed stepwise binary logistic regression analysis on the whole population, to analyse the relationship between the probability of an individual to develop chronic Chagas cardiomyopathy and the main covariates (sex and polymorphisms). Sex was considered as a binary covariate. In our stepwise binary logistic regression analysis, genotypes were considered as binary covariates. Indeed, for each polymorphism we had two alleles (A frequent one; a rare one). So, we obtained three genotypes (AA, Aa, and aa). In our stepwise binary logistic regression analysis, genotypes were considered as binary covariates. So, we performed three different analyses (Analysis 1: AA vs. Aa + aa (we supposed that the a allele is dominant); Analysis 2: AA + aa vs. Aa (we supposed that the heterozygote carriers are different from the homozygote ones); Analysis 3: AA + Aa vs. aa (we supposed that the A allele is dominant).

In multivariate analyses, several polymorphisms and gender were included as covariates. All the covariates are analyzed in the same time. In a stepwise approach, the worse associated covariate (non-significant) is removed and the analysis is run again up to keep only significant associated covariates.

An investigation of a candidate gene should test many SNPs individually for association. However, such multiple testing will increase the false-positive (type I error) rate under nominal significance thresholds (α = 0.05). So, we applied the Holm-Bonferroni sequential correction for multiple testing (39).

## Results

We conducted a study focusing on the *IL12B, IL10, IFNG*, and *IL4* genes. 35 Tag SNPs were selected (Supplementary Table 1). 97.8% markers were genotyped successfully on our cohort including ASY subjects (*n* = 118) and CCC patients (*n* = 315). The genotype distribution of each SNP is summarized in Supplementary Table 2. All the SNPs were in Hardy-Weinberg equilibrium on the ASY individuals considered as control subjects. Of the 118 ASY subjects, 45.3% were male, whereas in the CCC patients group, this percentage reaches 61.3%. The difference in sex distribution between the groups was significant (*p* = 1.21E-4; OR = 2.126; 95%CI: 1.450–3.12). It is well-known that male patients infected with T. cruzi have a higher risk of progression to CCC than female patients. Of 315 patients with CCC, 106 (42 men [39.6%] and 64 women [60.4%]) showed no significant ventricular dysfunction and were thus classified as having moderate CCC, whereas 199 (144 men [72.4%] and 55 women [27.6%]) had severe ventricular dysfunction and were classified as having severe CCC.

### Polymorphisms rs2546893G/A, rs919766A/C in and Around the *IL12B* Gene Are Associated to an Increased Risk of CCC

Fourteen on sixteen tag SNPs for the IL12B gene were genotyped successfully. *IL-12B* genotype data are shown in Tables 1, 2. All these markers are located in intronic region of IL12B gene. In the CCC subjects group, 269 (86.8%) subjects carried the rs2546893G/G or G/A genotypes whereas 79 (73.8%) of the ASY carried these genotypes (*p* = 0.002; OR = 1.548; 95%CI: 1.176–2.037 (see Table 1). These data suggest that rs2546893G/G or G/A genotypes are associated to an increased susceptibility to CCC.

**Table 1:** Association studies between CCC and ASY including as covariates the gender and the polymorphisms (genotype associations and allelic tests).

**GENE**	**Tag SNP**	**Genotype groups**	**Association test**
IL12B	rs2546890	GG vs. GA + AA	*p* = 0.103; OR = 1.231; 95%CI: 0.959–1.579
		MAF	ASY MAF(A) = 46.6%; CCC MAF(A) = 40.9%
		Allelic test	Chi-square = 2.11 and *p* = 0.15
	rs730691	GG vs. GA	*p* = 0.441; OR = 1.274; 95%CI: 0.688–2.358
		MAF	ASY MAF(A) = 9.1%; CCC MAF(A) = 11.1%
		Allelic test	Chi-square = 0.56 and *p* = 0.46
	rs2546893	GG + GA vs. AA	*p* = 0.002; OR = 1.548; 95%CI: 1.176–2.037
		MAF	ASY MAF(A) = 45.8%; CCC MAF(A) = 35.0%
		Allelic test	Chi-square = 7.89 and *p* = 0.005
	rs1003199	CC + CT vs. TT	*p* = 0.005; OR = 1.522; 95%CI: 1.134–2.045
		MAF	ASY MAF(T) = 43.1%; CCC MAF(T) = 35.9%
		Allelic test	Chi-square = 3.54 and *p* = 0.06
	rs3181216	TT vs. TA + AA	*p* = 0.702; OR = 1.047; 95%CI: 0.829–1.321
		MAF	ASY MAF(A) = 21.6%; CCC MAF(A) = 21.8%
		Allelic test	Chi-square = 0.001 and *p* = 0.97
	rs2569253	TT + TC vs. CC	*p* = 0.039; OR = 1.371; 95%CI: 1.015–1.852
		MAF	ASY MAF(C) = 40.7%; CCC MAF(C) = 35.3%
		Allelic test	Chi-square = 1.87 and *p* = 0.17
	rs2853694	AA vs. AC + CC	*p* = 0.382; OR = 1.105; 95%CI: 0.884–1.381
		MAF	ASY MAF(C) = 34.7%; CCC MAF(C) = 32.7%
		Allelic test	Chi-square = 0.28 and *p* = 0.60
	rs919766	AA vs. AC + CC	*p* = 0.001; OR = 1.653; 95%CI: 1.241–2.198
		MAF	ASY MAF(C) = 8.9%; CCC MAF(C) = 19.3%
		Allelic test	Chi-square = 36.67 and *p* < 0.00001
	rs2853696	GG vs. GA + AA	*p* = 0.981; OR = 1.003; 95%CI: 0.759–1.328
		MAF	ASY MAF(A) = 10.6%; CCC MAF(A) = 10.6%
		Allelic test	Chi-square = 0.002 and *p* = 0.097
	rs11574790	CC vs. CT + TT	*p* = 0.006; OR = 1.497; 95%CI: 1.124–1.996
		MAF	ASY MAF(T) = 8.3%; CCC MAF(T) = 16.6%
		Allelic test	Chi-square = 8.78 and *p* = 0.03
	rs3212227	AA vs. AC + CC	*p* = 0.973; OR = 1.004; 95%CI: 0.803–1.254
		MAF	ASY MAF(C) = 31.1%; CCC MAF(C) = 31.0%
		Allelic test	Chi-square = 0.001 and *p* = 0.97
	rs1368439	TT vs. TG + GG	*p* = 0.735; OR = 1.048; 95%CI: 0.800–1.373
		MAF	ASY MAF(G) = 11.2%; CCC MAF(G) = 10.5%
		Allelic test	Chi-square = 0.073 and *p* = 0.79
	rs6870828	AA vs. AG + GG	*p* = 0.754; OR = 1.040; 95%CI: 0.814–1.328
		MAF	ASY MAF(G) = 39.4%; CCC MAF(G) = 40.6%
		Allelic test	Chi-square = 0.086 and *p* = 0.77
	rs6859018	CC vs. CT + TT	*p* = 0.603; OR = 1.093; 95%CI: 0.782–1.527
		MAF	ASY MAF(T) = 33.0%; CCC MAF(T) = 32.5%
		Allelic test	Chi-square = 0.018 and *p* = 0.89
IL10	rs1800890	TT vs. TA + AA	*p* = 0.636; OR = 1.055; 95%CI: 0.845–1.318
		MAF	ASY MAF(A) = 26.4%; CCC MAF(A) = 24.7%
		Allelic test	Chi-square = 0.25 and *p* = 0.62
	rs1800896	AA + AG vs. GG	*p* = 0.013; OR = 1.527; 95%CI: 1.092–2.137
		MAF	ASY MAF(G) = 36.9%; CCC MAF(G) = 32.7%
		Allelic test	Chi-square = 1.24 and *p* = 0.27
	rs1800871	CC + TT vs. CT	*p* = 0.059; OR = 1.246; 95%CI: 0.992–1.566
		MAF	ASY MAF(T) = 35.3%; CCC MAF(T) = 35.3%
		Allelic test	Chi-square = 0.0001 and *p* = 0.99
	rs1518111	GG + AA vs. GA	*p* = 0.104; OR = 1.208; 95%CI: 0.962–1.516
		MAF	ASY MAF(A) = 32.4%; CCC MAF(A) = 34.2%
		Allelic test	Chi-square = 0.24 and *p* = 0.62
	rs3024496	TT + TC vs. CC	*p* = 0.031; OR = 1.437; 95%CI: 1.033–2.000
		MAF	ASY MAF(C) = 40.3%; CCC MAF(C) = 35.0%
		Allelic test	Chi-square = 1.89 and *p* = 0.17
	rs6673928	CC vs. CA	*p* = 0.329; OR = 2.141; 95%CI: 0.464–9.885
		MAF	ASY MAF(A) = 1.3%; CCC MAF(A) = 0.6%
		Allelic test	Chi-square = 0.95 and *p* = 0.33
IFNG	rs2069705	TT vs. TC + CC	*p* = 0.337; OR = 1.12; 95%CI: 0.89–1.41
		MAF	ASY MAF(C) = 39.6%; CCC MAF(C) = 37.9%
		Allelic test	Chi-square = 0.20 and *p* = 0.66
	rs1861494	AA vs. AG + GG	*p* = 0.057; OR = 1.24; 95%CI: 0.99–1.55
		MAF	ASY MAF(G) = 24.3%; CCC MAF(G) = 18.8%
		Allelic test	Chi-square = 3.21 and *p* = 0.07
	rs2069718	CC + CT vs. TT	*p* = 0.183; OR = 1.18; 95%CI: 0.92–1.52
		MAF	ASY MAF(C) = 48.2%; CCC MAF(C) = 52.4%
		Allelic test	Chi-square = 1.16 and *p* = 0.28
	rs2069727	AA + AG vs. GG	*p* = 0.232; OR = 1.22; 95%CI: 0.88–1.70
		MAF	ASY MAF(G) = 36.4%; CCC MAF(G) = 38.9%
		Allelic test	Chi-square = 0.45 and *p* = 0.50
	rs3181035	GG vs. GA + AA	*p* = 0.371; OR = 1.12; 95%CI: 0.88–1.42
		MAF	ASY MAF(A) = 17.0%; CCC MAF(A) = 13.7%
		Allelic test	Chi-square = 1.38 and *p* = 0.24
IL4	rs2070874	CC vs. CT + TT	*p* = 0.481; OR = 1.08; 95%CI: 0.87–1.34
		MAF	ASY MAF(T) = 27.0%; CCC MAF(T) = 27.9%
		Allelic test	Chi-square = 0.07 and *p* = 0.79
	rs2227284	AA + AC vs. CC	*p* = 0.268; OR = 1.15; 95%CI: 0.90–1.46
		MAF	ASY MAF(C) = 53.2%; CCC MAF(C) = 48.2%
		Allelic test	Chi-square = 1.62 and *p* = 0.20
	rs2243261	GG vs. GT + TT	*p* = 0.651; OR = 1.10; 95%CI: 0.74–1.63
		MAF	ASY MAF(T) = 3.8%; CCC MAF(T) = 4.6%
		Allelic test	Chi-square = 0.26 and *p* = 0.61
	rs2243268	AA vs. AC + CC	*p* = 0.467; OR = 1.08; 95%CI: 0.86–1.38
		MAF	ASY MAF(C) = 20.0%; CCC MAF(C) = 21.2%
		Allelic test	Chi-square = 0.15 and *p* = 0.70
	rs2243274	GG + GA vs. AA	*p* = 0.423; OR = 1.15; 95%CI: 0.82–1.60
		MAF	ASY MAF(A) = 31.2%; CCC MAF(A) = 35.8%
		Allelic test	Chi-square = 1.53 and *p* = 0.22
	rs2243290	CC + CA vs. AA	*p* = 0.352; OR = 1.21; 95%CI: 0.81–1.82
		MAF	ASY MAF(A) = 23.7%; CCC MAF(A) = 24.8%
		Allelic test	Chi-square = 0.12 and *p* = 0.73
	rs2406539	AA vs. AT + TT	*p* = 0.570; OR = 1.19; 95%CI: 0.65–2.17
		MAF	ASY MAF(T) = 7.5%; CCC MAF(T) = 9.2%
		Allelic test	Chi-square = 0.60 and *p* = 0.44

*MAF, Minor Allelic frequency*.

**Table 2:** Association studies between CCC with a left ventricular ejection fraction value under 0.4% and ASY including as covariates the gender and the polymorphism one by one.

**GENE**	**Tag SNP**		**Association tests**
IL12B	rs2546890	GG vs. GA + AA	*p* = 0.121; OR = 1.23; 95%CI: 0.95–1.61
		MAF	ASY MAF(A) = 46.6%; SEV CCC MAF(A) = 378%
		Allelic test	Chi-square = 4.30 and *p* = 0.04
	rs730691	GG vs. GA	*p* = 0.407; OR = 1.33; 95%CI: 0.68–2.58
		MAF	ASY MAF(A) = 9.1%; SEV CCC MAF(A) = 12.0%
		Allelic test	Chi-square = 0.91 and *p* = 0.34
	rs2546893	GG + GA vs. AA	*p* = 0.001; OR = 1.69; 95%CI: 1.23–2.34
		MAF	ASY MAF(A) = 45.8%; SEV CCC MAF(A) = 32.8%
		Allelic test	Chi-square = 9.84 and *p* = 0.002
	rs1003199	CC + CT vs. TT	*p* = 0.013; OR = 1.52; 95%CI: 1.09–2.13
		MAF	ASY MAF(T) = 43.123.7%; SEV CCC MAF(T) = 34.8%
		Allelic test	Chi-square = 3.98 and *p* = 0.05
	rs3181216	TT vs. TA + AA	*p* = 0.430; OR = 1.11; 95%CI: 0.86–1.43
		MAF	ASY MAF(A) = 23.7%; SEV CCC MAF(A) = 24.8%
		Allelic test	Chi-square = 0.80 and *p* = 0.37
	rs2569253	TT + CC vs. TC	*p* = 0.036; OR = 1.45; 95%CI: 1.02–2.05
		MAF	ASY MAF(C) = 40.7%; SEV CCC MAF(C) = 34.3%
		Allelic test	Chi-square = 2.33 and *p* = 0.13
	rs2853694	AA vs. AC + CC	*p* = 0.360; OR = 1.12; 95%CI: 0.88–1.43
		MAF	ASY MAF(C) = 34.7%; SEV CCC MAF(C) = 30.2%
		Allelic test	Chi-square = 1.34 and *p* = 0.25
	rs919766	AA vs. AC + CC	*p* = 0.007; OR = 1.49; 95%CI: 1.11–1.98
		MAF	ASY MAF(C) = 8.9%; SEV CCC MAF(C) = 20.7%
		Allelic test	Chi-square = 13.91 and *p* = 0.0002
	rs2853696	GG vs. GA + AA	*p* = 0.649; OR = 1.27; 95%CI: 0.45–3.56
		MAF	ASY MAF(A) = 10.6%; SEV CCC MAF(A) = 9.5%
		Allelic test	Chi-square = 0.21 and *p* = 0.64
	rs11574790	CC vs. CT + TT	*p* = 0.026; OR = 1.39; 95%CI: 1.04–1.86
		MAF	ASY MAF(T) = 8.3%; SEV CCC MAF(T) = 18.3%
		Allelic test	Chi-square = 10.98 and *p* = 0.0009
	rs3212227	AA vs. AC + CC	*p* = 0.631; OR = 1.06; 95%CI: 0.83–1.35
		MAF	ASY MAF(C) = 31.1%; SEV CCC MAF(C) = 31.9%
		Allelic test	Chi-square = 0.04 and *p* = 0.84
	rs1368439	TT vs. TG + GG	*p* = 0.547; OR = 1.09; 95%CI: 0.82–1.47
		MAF	ASY MAF(G) = 11.2%; SEV CCC MAF(G) = 9.9%
		Allelic test	Chi-square = 0.24 and *p* = 0.62
	rs6870828	AA + AG vs. GG	*p* = 0.181; OR = 1.28; 95%CI: 0.89–1.82
		MAF	ASY MAF(G) = 39.4%; SEV CCC MAF(G) = 42.4%
		Allelic test	Chi-square = 0.48 and *p* = 0.49
	rs6859018	CC vs. CT + TT	*P* = 0.898; OR = 1.02; 95%CI: 0.80–1.29
		MAF	ASY MAF(T) = 33.0%; SEV CCC MAF(T) = 34.3%
		Allelic test	Chi-square = 0.10 and *p* = 0.75
IL10	rs1800890	TT vs. TA + AA	*p* = 0.181; OR = 1.22; 95%CI: 0.91–1.63
		MAF	ASY MAF(A) = 26.4%; SEV CCC MAF(A) = 24.0%
		Allelic test	Chi-square = 0.44 and *p* = 0.51
	rs1800896	AA + AG vs. GG	*p* = 0.097; OR = 1.38; 95%CI: 0.94–2.01
		MAF	ASY MAF(G) = 36.9%; SEV CCC MAF(G) = 33.4%
		Allelic test	Chi-square = 0.74 and *p* = 0.39
	rs1800871	CC + TT vs. CT	*p* = 0.041; OR = 1.30; 95%CI: 1.01–1.66
		MAF	ASY MAF(T) = 35.3%; SEV CCC MAF(T) = 34.1%
		Allelic test	Chi-square = 6.18 and *p* = 0.01
	rs1518111	GG + AA vs. GA	*p* = 0.067; OR = 1.26; 95%CI: 0.98–1.61
		MAF	ASY MAF(A) = 32.4%; SEV CCC MAF(A) = 32.6%
		Allelic test	Chi-square = 0.001 and *p* = 0.97
	rs3024496	TT + TC vs. CC	*p* = 0.036; OR = 1.49; 95%CI: 1.03–2.16
		MAF	ASY MAF(C) = 40.3%; SEV CCC MAF(C) = 35.2%
		Allelic test	Chi-square = 1.55 and *p* = 0.21
	rs6673928	CC vs. CA	*p* = 0.432; OR = 2.12; 95%CI: 0.33–13.83
		MAF	ASY MAF(A) = 1.3%; SEV CCC MAF(A) = 0.5%
		Allelic test	Chi-square = 1.12 and *p* = 0.29
IFNG	rs2069705	TT vs. TC + CC	*p* = 0.333; OR = 1.13; 95%CI: 0.88–1.46
		MAF	ASY MAF(C) = 39.6%; SEV CCC MAF(C) = 37.1%
		Allelic test	Chi-square = 0.38 and *p* = 0.54
	rs1861494	AA vs. AG + GG	*p* = 0.067; OR = 1.26; 95%CI: 0.98–1.61
		MAF	ASY MAF(G) = 24.3%; SEV CCC MAF(G) = 17.0%
		Allelic test	Chi-square=4.88 and p=0.03
	rs2069718	CC + CT vs. TT	*p* = 0.147; OR = 1.23; 95%CI: 0.93–1.63
		MAF	ASY MAF(T) = 51.8%; SEV CCC MAF(T) = 46.7%
		Allelic test	Chi-square = 1.47 and *p* = 0.22
	rs2069727	AA + AG vs. GG	*p* = 0.254; OR = 1.23; 95%CI: 0.86–1.75
		MAF	ASY MAF(G) = 36.4%; SEV CCC MAF(G) = 39.5%
		Allelic test	Chi-square = 0.60 and *p* = 0.44
	rs3181035	GG vs. GA + AA	*p* = 0.440; OR = 1.11; 95%CI: 0.85–1.45
		MAF	ASY MAF(A) = 17.0%; SEV CCC MAF(A) = 13.4%
		Allelic test	Chi-square = 1.46 and *p* = 0.23
**IL4**	rs2070874	CC vs. CT + TT	*p* = 0.274; OR = 1.14; 95%CI: 0.90–1.45
		MAF	ASY MAF(T) = 27.0%; SEV CCC MAF(T) = 30.8%
		Allelic test	Chi-square = 1.07 and *p* = 0.30
	rs2227284	AA + AC vs. CC	*p* = 0.059; OR = 1.30; 95%CI: 0.99–1.72
		MAF	ASY MAF(C) = 53.2%; SEV CCC MAF(C) = 43.3%
		Allelic test	Chi-square = 5.48 and *p* = 0.02
	rs2243261	GG vs. GT + TT	*p* = 0.841; OR = 1.05; 95%CI: 0.68–1.62
		MAF	ASY MAF(T) = 3.8%; SEV CCC MAF(T) = 4.8%
		Allelic test	Chi-square = 0.34 and *p* = 0.56
	rs2243268	AA vs. AC + CC	*p* = 0.159; OR = 1.20; 95%CI: 0.93–1.55
		MAF	ASY MAF(C) = 20.0%; SEV CCC MAF(C) = 23.8%
		Allelic test	Chi-square = 1.20 and *p* = 0.27
	rs2243274	GG + GA vs. AA	*p* = 0.225; OR = 1.25; 95%CI: 0.87–1.80
		MAF	ASY MAF(A) = 31.2%; SEV CCC MAF(A) = 38.4%
		Allelic test	Chi-square = 3.16 and *p* = 0.08
	rs2243290	CC + CA vs. AA	*p* = 0.842; OR = 1.04; 95%CI: 0.68–1.61
		MAF	ASY MAF(A) = 23.7%; SEV CCC MAF(A) = 27.2%
		Allelic test	Chi-square = 0.91 and *p* = 0.34
	rs2406539	AA vs. AT + TT	*p* = 0.538; OR = 1.23; 95%CI: 0.64–2.35
		MAF	ASY MAF(T) = 7.5%; SEV CCC MAF(T) = 10.0%
		Allelic test	Chi-square = 1.05 and *p* = 0.30

These association tests are genotype association and allelic tests.

*SEV CCC, Severe CCC*.

For the rs919766A/C polymorphism, 107 (34.7%) CCC subjects carried the C/C or A/C genotypes vs. 17 (15.9%) for the ASY group (*p* = 0.001; OR = 1.653; 95%CI: 1.241–2.198). For the rs1003199C/T polymorphism, 276 (88.7%) CCC subjects carried the C/C or C/T genotypes vs. 285 (78.7%) for the ASY group. This difference was significant (*p* = 0.005; OR = 1.522; 95%CI: 1.134–2.045). For the rs11574790C/T polymorphism, 95 (30.5%) CCC subjects carried the C/T or T/T genotypes vs. 17 (15.7%) for the ASY group (*p* = 0.006; OR = 1.497; 95%CI: 1.124–1.996). For the rs2569253T/C polymorphism, 256 (87.46%) CCC subjects carried the T/T or T/C genotypes vs. 81 (79.4%) for the ASY (*p* = 0.039; OR = 1.371; 95%CI: 1.015–1.852). After performing a correction for multiple testing using the Bonferroni methods, the two main polymorphisms (rs919766 and rs25466893) remain significant (corrected *p*-values were 0.014 and 0.026, respectively).

In a multivariate analysis, including these five polymorphisms and gender as covariates, the gender (*p* = 0.004; OR = 2.036; 95% CI: 1.249–3.318) and two markers (rs2546893: *p* = 0.005; OR = 1.536; 95% CI: 1.139–2.070; rs919766: *p* = 0.014; OR = 1.477; 95% CI: 1.081–2.020) remained significantly associated. The three other markers were excluded from the best model. Allelic tests confirmed the association of these two IL12 markers. The rs919766C allele (*p* < 0.00001) and the rs2546893G allele (*p* = 0.005) were associated to an increase risk to CCC development.

The same analysis was performed between the ASY subjects and severe CCC patients. The same polymorphisms were associated, in univariate analysis, to an increased risk to develop severe chronic cardiomyopathy (rs2546893G/A: *p* = 0.001; OR = 1.706; 95%CI: 1.236–2.353; rs919766A/C: *p* = 0.001; OR = 1.672; 95%CI: 1.231–2.267; rs1003199C/T: *p* = 0.013; OR = 1.531; 95%CI: 1.236–2.353; rs2569253T/C: *p* = 0.037; OR = 1.445; 95%CI: 1.022–2.041; rs11574790C/T: *p* = 0.004; OR = 1.562; 95%CI: 1.151–2.123) (see Table 2). After correction for multiple testing the polymorphism rs2546893 remains significant (corrected *p*-value = 0.014) and the polymorphism rs919766 is borderline (corrected *p*-value = 0.091). In a multivariate analysis, the gender and the two polymorphisms remained significantly associated (rs2546893G/A: *p* = 0.005; OR = 1.667; 95% CI: 1.170–2.375; rs919766A/C: *p* = 0.024; OR = 1.471; 95%CI: 1.051–2.058; gender: *p* = 2.25 × 10^−5^; OR = 3.261; 95% CI: 1.888–5.632). the allelic tests confirmed that the rs2546893G allele (*p* < 0.002) and the rs919766C allele (*p* = 0.0002) were associated to an increase risk to severe CCC. None of the studied *IL12B* markers discriminated moderate from severe CCC patient groups.

### Polymorphism rs3024496T/C Around the *IL10* Gene Is Showing a Trend of Association to CCC

Six tag SNPs were genotyped for the *IL10* gene. Table 1 shows comparison between CCC and ASY, while Table 2 shows comparison of severe CCC and ASY. For the rs1800896A/G polymorphism, 282 (91.9%) CCC subjects carried the A/A or A/G genotypes vs. 90 (84.1%) for the ASY (*p* = 0.013; OR = 1.527; 95%CI: 1.092–2.137). The same result was obtained when the analysis was restricted to severe CCC vs. ASY (*p* = 0.028; OR = 1.536; 95%CI: 1.047–2.252) (see Table 2). For the rs3024496T/C polymorphism, 284 (91.3%) CCC subjects carried the T/T or T/C genotypes vs. 92 (84.3%) for the ASY (*p* = 0.031; OR = 1.437; 95%CI: 1.033–2.000) (see Table 1). The same result was obtained when the analysis was restricted to severe CCC vs. ASY (*p* = 0.049; OR = 1.462; 95%CI: 1.002–2.132) (see Table 2). For the rs1800871 marker, we found a significant association when severe CCC patients were compared the ASY subjects (*p* = 0.035; OR = 1.311; 95%CI: 1.019–1.686). No association was found for this last marker in the comparison between ASY and the whole CCC group (see Table 1). After correction for multiple testing, the polymorphism rs1800896A/G and rs3024496T/C has shown only trends of association (rs1800896A/G: corrected *p*-value = 0.078). The allelic tests reach the same trends of association (rs3024496C *p* = 0.17 and rs1800896G *p* = 0.27).

Based on the results of the multivariate analysis, we had an association between CCC and ASY with two SNPs located in the IL12B gene (rs2546893 and rs919766) and one polymorphism in the IL10 gene (rs3024496). Subjects carrying the three susceptibility genotypes [rs2546893(GG or GA) + rs919766(CC or AC) + rs3024496(TT or TC)] have a higher risk (OR = 2.68) to develop CCC. In contrast, subjects carrying the resistant genotypes (rs2546893AA + r919766AA + rs3024496CC) have a lower risk [OR = 4.48 (1/0.223)] to develop CCC (Table 3).

**Table 3:** Genotype combination analysis based on the following polymorphisms (rs2546893/rs919766/rs3024496).

**Genotype** **combinations**	**IL12** **rs2546893**	**IL12** **rs919766**	**IL10** **rs3024496**	**CCC**	**ASY**	**Total**	**% in ASY**	**% in CCC**	**Ratio CCC/ASY**	***p*-value**	**Odds Ratio**	**95% C.I. for EXP(B)**
1 S1 S2 S3	GG + GA	CC + AC	TC + CC	89	14	103	**14%**	**86%**	**6.36**	0.0006	2.677	1.468–4.838
2 S1 S2 R3	GG + GA	CC + AC	CC	9	3	12	**25%**	**75%**	**3.00**	0.6340	1.028	0.2758
3 S1 R2 S3	GG + GA	AA	TC + CC	149	46	195	**24%**	**76%**	**3.24**	0.1963	1.217	0.7777–1.928
4 R1 S2 S3	AA	AC + CC	TC + CC	6	0	6	**0%**	**100%**	**X**	0.1688	X	X
5 S1 R2 R3	GG + GA	AA	CC	13	11	24	**46%**	**54%**	**1.18**	0.0058	0.3483	0.1434–0.7776
6 R1 S2 R3	AA	AC + CC	CC	1	0	1	**0%**	**100%**	**X**	0.7450	X	X
7 R1 R2 S3	AA	AA	TC + CC	29	25	54	**46%**	**54%**	**1.16**	<0.0001	0.3320	0.1834–0.6135
8 R1 R2 R3	AA	AA	CC	2	3	5	**60%**	**40%**	**0.67**	0.1075	0.2230	0.0393–1.110

R, Resistant genotype; S, Susceptibility genotype; SNP 1 = rs2546893/SNP2 = rs919766/SNP3 = rs3024496.

Bold value indicates % ASY, Percentage of ASY subjects carrying the genotype combination; % CCC, Percentage of CCC subjects carrying the genotype combination Ratio; CCC/ASY, Ratio between these two percentage.

### No Clear Evidence of Association Was Detected for Tag Polymorphisms in the *IFNG* and *IL4* Genes

Six tag SNPs were genotyped for the *IFNG* gene. For the rs1861494 A/G polymorphism, a trend of association was detected. Indeed, 202 (64.7%) CCC subjects carried the A/A genotype vs. 62 (54.9%) for the ASY (*p* = 0.057; OR = 1.24; 95%CI: 0.99–1.55) (see Supplementary Table 2). The same trend appeared on severe cases (*p* = 0.067; OR = 1.26; 95%CI: 0.98–1.61) (see Table 2). Seven tag SNPs into the *IL4* gene were genotyped on our whole study population (see Supplementary Table 2). Comparisons between ASY and CCC patients disclosed no significant associations (see Tables 1, 2).

## Discussion

Association studies of cytokine gene polymorphisms in Chagas disease frequently include a small number of patients, and are often not replicated in different studies or ethnical groups. The present study was designed to determine whether SNPs in *IL12B, IL10, IFNG* and *IL4*, key genes involved in the promotion and control of Th1 differentiation and IFN-g production to *T. cruzi*, are associated with Chagas disease outcome. For that, we used a larger population and performed a comprehensive assessment of 35 Tag SNPs covering all the genetic information of the mentioned genes. We found significant associations of genotypes and alleles of *IL12B* and *IL10* with increased risk to CCC progression.

For the *IL12B* gene region, in a multivariate analysis, two SNPs were significantly associated to disease (rs2546893G/A, rs919766A/C). The rs919766C and rs2546893G alleles were associated to an increase risk to CCC development. Interestingly, these two markers are located 5 to 13 kb away from the rs3212227 polymorphism (*IL12B* 3′UTR region), that was previously associated to Chagas cardiomyopathy (31). Our results are consistent as these 3 markers are in linkage disequilibrium (*D*′ = 0.99) on the African (Yoruba) reference population and the markers rs919766 and rs3212227 are in strong linkage disequilibrium (*D*′ = 0.99) on the European reference population.

For the *IL10* gene, the rs3024496 polymorphism (located in the 3′UTR region), and rs1800896 polymorphism (located in the promoter region) have shown trends of association to susceptibility to cardiomyopathy. The rs3024496C and rs1800896G alleles were more frequent in patients. Our data are consistent with the previous study that showed that the marker (IL10-1082 known as rs1800896 too) was previously associated with development of CCC (33). Costa et al. have shown that the A allele has been associated with lower IL-10 expression in disease and lower left ventricular ejection fraction values in CCC (33). The two markers (rs3024496 and rs1800896) are in strong linkage disequilibrium on the European reference population (*D*′ = 1) and on the Yoruba population (*D*′ = 0.83).

Three SNPs in the *IL10* gene (the two rs3024496, rs1800896 mentioned above, plus rs1800871) detected trends of association. A study performed on a Brazilian population infected by *Leishmania braziliensis* has shown that these markers are correlated (*r*^2^ value >0.8) (40). So, from one Brazilian sub population to another one, the associated marker may change (due to light allelic frequency variation) or population stratification. However, the associated marker is coming from the same correlation block.

We did not find any significant association with SNPs from the *IFNG* and *IL4* genes. This is in agreement with Arnez et al. who studied some of these same SNPs (rs2070874, rs2227284, rs2243268, rs2243274, rs2243290 in an Bolivian population (29), and Flórez et al. who studied one SNP not tested by our group (rs2243250) in a Colombian population, failing to find any association (30). Taken together, these results suggest that we do not have evidence that polymorphisms in or within the IFN gene control the expression of this key cytokine for the pathogenic process.

During *T. cruzi* infection, once the inflammatory process starts, IFN-γ will be produced by Th1 cells and act as a prime inflammatory cytokine in different pathways of the immune system, such as upregulating MHC class I and class II molecules, suppressing Th2 immune responses by antagonism of IL-4 production, inducing high levels of antigen presentation and activating macrophages (41). Moreover, CCC patients have an increased peripheral production of IFN-γ and TNF-α when compared to patients with the asymptomatic/indeterminate form. On the other hand, IFN-γ has direct effects on cardiomyocytes and perhaps other cells of the myocardium (34). At this level we can speculate that the existence of functional or pathogenic variants in or within the IFN gene will have a too strong deleterious effect that will be lethal. So, the genetic control of the human susceptibility to cardiomyopathy mainly target genes that are involved in the regulation of this TH1 response, such as *IL12B* and *IL10* genes.

On the other hand, our data confirmed the involvement of *IL12B* and *IL10* in the control of susceptibility to human Chagas cardiomyopathy. The *IL10* polymorphism has been described as a functional polymorphism. It remains to be tested whether the *IL12B* polymorphism and linked SNPs are functional as well. Taking into account the effects of both cytokines on Th1 T cell differentiation and IFNγ production, it can be hypothesized that this is linked to the increased number of IFNγ producing Th1 T cells in the peripheral blood and heart tissue of CCC patients. Our study and others have identified several genes in the control of human susceptibility to chronic disease. However, each gene is characterized by a low odds ratio, implying a small contribution in Chagas disease infection. However, the combination of the several genetic markers increases the protection from disease development, with individual odds ratio around 1.5, to ca. 4.5 in patients carrying all the protection genotypes in all three loci. The identification of more genetic markers for CCC will provide information for pathogenesis as well as therapeutic targets. Identifying early or causal predictive genetic factors for the clinical progression of the disease is essential for clarifying the pathogenesis and defining appropriate treatment modalities.

## Data Availability Statement

The datasets presented in this study can be found in online repositories. The names of the repository/repositories and accession number(s) can be found in the article/Supplementary Material.

## Ethics Statement

The studies involving human participants were reviewed and approved by The INSERM Internal Review Board, The Brazilian National Ethics in Research Commission (CONEP). The patients/participants provided their written informed consent to participate in this study.

## Author Contributions

JK, EC-N, and CC: study design. AF-B and SC: experiments. AF-B, BI, CP, BS, HL-W, PB, JM-N, AS, FD, MH, MS, AF, AP, ED, VR, JK, CC, and EC-N: manuscript preparation. All authors contributed to the article and approved the submitted version.

## Conflict of Interest

The authors declare that the research was conducted in the absence of any commercial or financial relationships that could be construed as a potential conflict of interest.
