# Corrigendum: Polymorphisms in Genes Affecting Interferon-γ Production and Th1 T Cell Differentiation Are Associated With Progression to Chagas Disease Cardiomyopathy

**DOI:** 10.3389/fimmu.2020.593759

**Published:** 2020-09-10

**Authors:** Amanda Farage Frade-Barros, Barbara Maria Ianni, Sandrine Cabantous, Cristina Wide Pissetti, Bruno Saba, Hui Tzu Lin-Wang, Paula Buck, José Antonio Marin-Neto, André Schmidt, Fabrício Dias, Mario Hiroyuki Hirata, Marcelo Sampaio, Abílio Fragata, Alexandre Costa Pereira, Eduardo Donadi, Virmondes Rodrigues, Jorge Kalil, Christophe Chevillard, Edecio Cunha-Neto

**Affiliations:** ^1^Heart Institute (InCor), University of São Paulo School of Medicine (FMUSP), São Paulo, Brazil; ^2^Institute for Investigation in Immunology (iii), INCT, São Paulo, Brazil; ^3^Aix-Marseille Université, INSERM, GIMP UMR_S906, Marseille, France; ^4^Division of Clinical Immunology and Allergy, University of São Paulo School of Medicine, São Paulo, Brazil; ^5^Bioengineering Program, Instituto Tecnológico, Universidade Brasil, São Paulo, Brazil; ^6^Laboratory of Immunology, Universidade Federal Do Triângulo Mineiro (UFTM), Uberaba, Brazil; ^7^Laboratório de Investigação Molecular em Cardiologia, Instituto de Cardiologia Dante Pazzanese (IDPC), São Paulo, Brazil; ^8^School of Medicine of Ribeirão Preto (FMRP), University of São Paulo, Ribeirão Preto, Brazil; ^9^Department of Clinical and Toxicological Analyses, Faculty of Pharmaceutical Sciences, University of São Paulo (USP), São Paulo, Brazil; ^10^Aix Marseille Université, INSERM, TAGC Theories and Approaches of Genomic Complexity, UMR_1090, Marseille, France

**Keywords:** Chagas disease, cardiomyopathy, susceptibility, IL12, IL 10, IFN, IL4

In the original article, we neglected to include the funding from the National Institute of Health (P50 AI098461-02 and U19AI098461-06) to EC-N.

The authors apologize for this error and state that this does not change the scientific conclusions of the article in any way. The original article has been updated.

