# Supplementary material for: Polymorphisms in Genes Affecting Interferon-γ Production and Th1 T Cell Differentiation Are Associated With Progression to Chagas Disease Cardiomyopathy
Source: Front Immunol. 2020 Jul 7;11:1386. doi: 10.3389/fimmu.2020.01386 (PMC7358543; doi:10.3389/fimmu.2020.01386)
Supplement: Supplementary file 1 [file Table_1.docx]

**Supplementary table 1:** List of the tag SNPs genotyped on the original study population

| **GENE** | **Tag SNP** | | **Position relative**  **to coordinate system** | | | **Position relative to traduction start point** |  |
| --- | --- | --- | --- | --- | --- | --- | --- |
|  |  | |  | | |  |  |
| **IL12B** | rs1422876 G/A | | 158761299 | | | -7509 |  |
|  | rs2546890 G/A | | 158759900 | | | -6110 |  |
|  | rs730691 G/A | | 158756227 | | | -2437 |  |
|  | rs2546893 G/A | | 158755960 | | | -2170 |  |
|  | rs1003199 C/T | | 158755566 | | | -1776 |  |
|  | rs3181216 T/A | | 158752978 | | | +812 |  |
|  | rs2569253 T/C | | 158750993 | | | +2797 |  |
|  | rs2853694 A/C | | 158749088 | | | +4702 |  |
|  | rs919766 A/C | | 158747565 | | | +6225 |  |
|  | rs2853696 G/A | | 158744660 | | | +9130 |  |
|  | rs2195940 G/A | | 158744352 | | | +9438 |  |
|  | rs11574790 C/T | | 158743846 | | | +9944 |  |
|  | rs3212227 A/C | | 158742950 | | | +10840 |  |
|  | rs1368439 T/G | | 158742014 | | | +11776 |  |
|  | rs6870828 G/A | | 158738512 | | | +15278 |  |
|  | rs6859018 C/T | | 158736992 | | | +16798 |  |
|  |  | |  | | |  |  |
| **IL10** | rs1800890 T/A | | 206949365 | | | -3585 |  |
|  | rs1800896 A/G | | 206946897 | | | -1117 |  |
|  | rs1800871 C/T | | 206946634 | | | -854 |  |
|  | rs1518111 G/A | | 206944645 | | | +1135 |  |
|  | rs3024496 T/C | | 206941864 | | | +3916 |  |
|  | rs6673928 C/A | | 206937245 | | | +8535 |  |
|  | |  | |  |  | | |
| **IFNG** | | rs2069705 T/C | | 68555011 | -1616 | | |
|  | | rs1861494 A/G | | 68551409 | +1986 | | |
|  | | rs2069718 C/T | | 68550162 | +3233 | | |
|  | | rs2069727 A/G | | 68548223 | +5172 | | |
|  | | rs3181035 G/A | | 68546396 | +6999 | | |
|  | | rs1236947 A/G | | 68544896 | +8499 | | |
|  | |  | |  |  | | |
| **IL4** | | rs2070874 C/T | | 132009710 | -33 | | |
|  | | rs2227284 T/G | | 132012725 | +2982 | | |
|  | | rs2243261 G/T | | 132012806 | +3063 | | |
|  | | rs2243268 A/C | | 132013963 | +4220 | | |
|  | | rs2243274 G/A | | 132014832 | +5089 | | |
|  | | rs2243290 C/A | | 132018169 | +8426 | | |
|  | | rs2406539 T/A | | 132023276 | +13533 | | |
