# Supplementary material for: Polymorphisms in Genes Affecting Interferon-γ Production and Th1 T Cell Differentiation Are Associated With Progression to Chagas Disease Cardiomyopathy
Source: Front Immunol. 2020 Jul 7;11:1386. doi: 10.3389/fimmu.2020.01386 (PMC7358543; doi:10.3389/fimmu.2020.01386)
Supplement: Supplementary file 2 [file Table_2.docx]

**Supplementary table 2:** Genotype distribution on controls (asymptomatic individuals) and cases (CCC) taking into account the gender and the left ventricular ejection fraction values.

| **Gene** | **SNP** | | **ASY** | | | **CCC** | | | **SEV CCC**  **(EF<0.4)** | | | **MOD CCC**  **(EF>0.4)** | | |
| --- | --- | --- | --- | --- | --- | --- | --- | --- | --- | --- | --- | --- | --- | --- |
|  |  |  | **Total** | Male | Female | **Total** | Male | Female | **Total** | Male | Female | **Total** | Male | Female |
| **IL12B** | **rs2546890** | **GG** | **29**  **(27.9%)** | 14  (30.4%) | 14  (24.6%) | **116**  **(37.6%)** | 79  (43.2%) | 36  (29.8%) | **76**  **(40.2%)** | 59  (43.7%) | 17  (31.5%) | **34**  **(531.5%)** | 15  (35.7%) | 19  (28.8%) |
|  |  | **GA** | **53**  **(51.0%)** | 22  (47.8%) | 31  (54.4%) | **131**  **(42.8%)** | 75  (41.0%) | 55  (45.5%) | **83**  **(43.9%)** | 55  (40.7%) | 28  (51.9%) | **45**  **(41.7%)** | 19  (45.2%) | 26  (39.4%) |
|  |  | **AA** | **22**  **(20.0%)** | 10  (21.7%) | 12  (21.1%) | **60**  **(19.6%)** | 29  (15.8%) | 30  (24.8%) | **30**  **(15.9%)** | 21  (15.6%) | 9  (16.7%) | **29**  **(26.9%)** | 8  (19.0%) | 21  (31.8%) |
|  |  |  |  |  |  |  |  |  |  |  |  |  |  |  |
| **IL12B** | **rs730691** | **GG** | **76**  **(81.7%)** | 36  (81.8%) | 39  (81.3%) | **178**  **(77.7%)** | 108  (77.7%) | 69  (77.5%) | **105**  **(76.1%)** | 74  (76.3%) | 31  (75.6%) | **69**  **(82.1%)** | 32  (84.2%) | 37  (80.4%) |
|  |  | **GA** | **17**  **(16.3%)** | 8  (18.2%) | 9  (18.8%) | **51**  **(22.3%)** | 31  (22.3%) | 20  (22.5%) | **33**  **(23.9%)** | 23  (23.7%) | 10  (24.4%) | **15**  **(17.9%)** | 6  (15.8%) | 9  (19.6%) |
|  |  |  |  |  |  |  |  |  |  |  |  |  |  |  |
| **IL12B** | **rs2546893** | **GG** | **37**  **(34.6%)** | 16  (32.7%) | 20  (35.1%) | **134**  **(43.2%)** | 89  (47.6%) | 45  (37.2%) | **87**  **(46.0%)** | 66  (48.5%) | 21  (39.6%) | **42**  **(37.5%)** | 18  (40.9%) | 24  (35.3%) |
|  |  | **GA** | **42**  **(39.3%)** | 20  (40.8%) | 22  (38.6%) | **135**  **(43.5%)** | 71  (38.0%) | 62  (51.2%) | **80**  **(42.3%)** | 52  (38.2%) | 28  (52.8%) | **52**  **(46.4%)** | 18  (40.9%) | 34  (50.0%) |
|  |  | **AA** | **28**  **(26.2%)** | 13  (26.5%) | 15  (26.3%) | **41**  **(13.2%)** | 27  (14.4%) | 14  (11.6%) | **22**  **(11.6%)** | 18  (13.2%) | 4  (7.5%) | **18**  **(16.1%)** | 8  (18.2%) | 10  (14.7%) |
|  |  |  |  |  |  |  |  |  |  |  |  |  |  |  |
| **IL12B** | **rs1003199** | **CC** | **38**  **(35.2%)** | 16  (32.0%) | 21  (36.8%) | **123**  **(39.5%)** | 80  (42.8%) | 43  (35.2%) | **80**  **(41.9%)** | 60  (43.8%) | 20  (37.0%) | **36**  **(33.0%)** | 14  (32.6%) | 22  (33.3%) |
|  |  | **CT** | **47**  **(43.5%)** | 22  (44.0%) | 25  (43.9%) | **153**  **(49.2%)** | 81  (43.3%) | 70  (57.4%) | **89**  **(46.6%)** | 59  (43.1%) | 30  (55.6%) | **61**  **(56.0%)** | 22  (51.2%) | 39  (59.1%) |
|  |  | **TT** | **23**  **(21.3%)** | 12  (24.0%) | 11  (19.3%) | **35**  **(11.3%)** | 26  (13.9%) | 9  (7.4%) | **22**  **(11.5%)** | 18  (13.1%) | 4  (7.4%) | **12**  **(11.0%)** | 7  (16.3%) | 5  (7.6%) |
|  |  |  |  |  |  |  |  |  |  |  |  |  |  |  |
| **IL12B** | **rs3181216** | **TT** | **61**  **(58.7%)** | 28  (60.9%) | 32  (56.1%) | **177**  **(61.7%)** | 115  (67.6%) | 62  (53.9%) | **118**  **(66.3%)** | 86  (68.8%) | 32  (30.4%) | **53**  **(53.5%)** | 24  (61.5%) | 29  (48.3%) |
|  |  | **TA** | **41**  **(39.4%)** | 18  (39.1%) | 23  (40.4%) | **95**  **(33.1%)** | 46  (27.1%) | 47  (40.9%) | **54**  **(30.3%)** | 35  (28.0%) | 19  (35.8%) | **37**  **(37.4%)** | 10  (25.6%) | 27  (45.0%) |
|  |  | **AA** | **2**  **(1.9%)** | 0  (0.0%) | 2  (3.5%) | **15**  **(5.2%)** | 9  (5.3%) | 6  (5.2%) | **6**  **(3.4%)** | 4  (3.2%) | 2  (3.8%) | **9**  **(9.1%)** | 5  (12.8%) | 4  (6.7%) |
|  |  |  |  |  |  |  |  |  |  |  |  |  |  |  |
| **IL12B** | **rs2569253** | **TT** | **40**  **(39.2%)** | 14  (32.6%) | 25  (43.1%) | **123**  **(42.0%)** | 77  (44.0%) | 46  (39.7%) | **78**  **(43.1%)** | 57  (44.5%) | 21  (39.6%) | **38**  **(37.3%)** | 14  (34.1%) | 34  (39.3%) |
|  |  | **TC** | **41**  **(40.2%)** | 20  (46.5%) | 21  (36.2%) | **133**  **(45.4%)** | 73  (41.7%) | 58  (50.0%) | **82**  **(45.3%)** | 55  (43.0%) | 27  (50.9%) | **48**  **(47.1%)** | 18  (43.9%) | 30  (49.2%) |
|  |  | **CC** | **21**  **(20.6%)** | 9  (20.9%) | 12  (20.7%) | **37**  **(12.6%)** | 25  (14.3%) | 12  (10.3%) | **21**  **(11.6%)** | 16  (12.5%) | 5  (9.4%) | **16**  **(15.7%)** | 9  (22.0%) | 7  (11.5%) |
|  |  |  |  |  |  |  |  |  |  |  |  |  |  |  |
| **IL12B** | **rs2853694** | **AA** | **46**  **(41.4%)** | 22  (44.0%) | 23  (38.3%) | **147**  **(47.0%)** | 98  (51.6%) | 49  (40.5%) | **92**  **(47.4%)** | 70  (49.6%) | 22  (41.5%) | **48**  **(44.0%)** | 22  (51.2%) | 26  (39.4%) |
|  |  | **AC** | **53**  **(47.7%)** | 23  (46.0%) | 30  (50.0%) | **127**  **(40.6%)** | 73  (38.4%) | 53  (43.8%) | **87**  **(44.6%)** | 60  (42.6%) | 27  (50.9%) | **38**  **(34.9%)** | 13  (30.2%) | 25  (37.9%) |
|  |  | **CC** | **12**  **(10.9%)** | 5  (10.0%) | 7  (11.7%) | **39**  **(12.5%)** | 19  (10.0%) | 19  (15.7%) | **15**  **(7.7%)** | 11  (7.8%) | 4  (7.5%) | **23**  **(21.1%)** | 8  (18.6%) | 15  (22.7%) |
|  |  |  |  |  |  |  |  |  |  |  |  |  |  |  |
| **IL12B** | **rs919766** | **AA** | **90**  **(84.1%)** | 40  (81.6%) | 50  (86.2%) | **201**  **(65.3%)** | 116  (62.4%) | 83  (69.2%) | **121**  **(63.4%)** | 84  (60.9%) | 37  (69.8%) | **74**  **(68.5%)** | 29  (69.0%) | 45  (68.2%) |
|  |  | **AC** | **15**  **(14.0%)** | 8  (16.3%) | 7  (12.1%) | **95**  **(30.8%)** | 60  (32.3%) | 35  (29.2%) | **61**  **(31.9%)** | 45  (32.6%) | 16  (30.2%) | **32**  **(29.6%)** | 13  (31.0%) | 19  (28.8%) |
|  |  | **CC** | **2**  **(1.9%)** | 1  (2.0%) | 1  (1.7%) | **12**  **(3.9%)** | 10  (5.4%) | 2  (1.7%) | **9**  **(4.7%)** | 9  (6.5%) | 0  (0.0%) | **2**  **(1.9%)** | 0  (0.0%) | 2  (3.0%) |
|  |  |  |  |  |  |  |  |  |  |  |  |  |  |  |
| **IL12B** | **rs2853696** | **GG** | **86**  **(79.6%)** | 39  (83.0%) | 46  (76.7%) | **249**  **(80.8%)** | 158  (85.9%) | 90  (73.8%) | **156**  **(82.1%)** | 115  (84.6%) | 41  (75.9%) | **85**  **(78.7%)** | 37  (88.1%) | 48  (72.7%) |
|  |  | **GA** | **21**  **(19.4%)** | 7  (14.9%) | 14  (23.3%) | **53**  **(17.2%)** | 23  (12.5%) | 29  (23.8%) | **32**  **(16.8%)** | 20  (14.7%) | 12  (22.2%) | **19**  **(17.6%)** | 3  (7.1%) | 16  (24.2%) |
|  |  | **AA** | **1**  **(0.9%)** | 3  (1.6%) | 0  (0.0%) | **6**  **(1.9%)** | 3  (1.6%) | 3  (2.5%) | **2**  **(1.1%)** | 1  (0.7%) | 1  (1.9%) | **4**  **(3.7%)** | 2  (4.8%) | 2  (3.0%) |
|  |  |  |  |  |  |  |  |  |  |  |  |  |  |  |
| **IL12B** | **rs11574790** | **CC** | **91**  **(84.3%)** | 39  (79.6%) | 51  (87.9%) | **216**  **(69.5%)** | 126  (67.0%) | 88  (72.7%) | **126**  **(66.0%)** | 87  (63.5%) | 39  (72.2%) | **83**  **(75.5%)** | 35  (100%) | 48  (72.7%) |
|  |  | **CT** | **16**  **(14.8%)** | 10  (20.4%) | 6  (10.3%) | **87**  **(28.0%)** | 56  (29.8%) | 31  (25.6%) | **60**  **(31.4%)** | 45  (32.8%) | 15  (27.8%) | **25**  **(22.7%)** | 0  (0.0%) | 16  (24.2%) |
|  |  | **TT** | **1**  **(0.9%)** | 0  (0.0%) | 1  (1.7%) | **8**  **(2.6%)** | 6  (3.2%) | 2  (1.7%) | **5**  **(2.6%)** | 5  (3.6%) | 0  (0.0%) | **2**  **(1.8%)** | 0  (0.0%) | 2  (3.0%) |
|  |  |  |  |  |  |  |  |  |  |  |  |  |  |  |
| **IL12B** | **rs3212227** | **AA** | **52**  **(49.1%)** | 27  (55.1%) | 25  (43.9%) | **152**  **(49.4%)** | 89  (48.1%) | 62  (51.2%) | **93**  **(48.7%)** | 65  (47.4%) | 28  (51.9%) | **56**  **(52.8%)** | 22  (53.7%) | 34  (52.3%) |
|  |  | **AC** | **42**  **(39.6%)** | 19  (38.8%) | 23  (40.4%) | **121**  **(39.3%)** | 71  (38.4%) | 49  (40.5%) | **74**  **(38.7%)** | 54  (39.4%) | 20  (37.0%) | **42**  **(39.6%)** | 15  (36.6%) | 27  (41.5%) |
|  |  | **CC** | **12**  **(11.3%)** | 3  (6.1%) | 9  (15.8%) | **35**  **(11.4%)** | 25  (13.5%) | 10  (8.3%) | **24**  **(12.6%)** | 18  (13.1%) | 6  (11.1%) | **8**  **(7.5%)** | 4  (9.8%) | 4  (6.2%) |
|  |  |  |  |  |  |  |  |  |  |  |  |  |  |  |
| **IL12B** | **rs1368439** | **TT** | **88**  **(78.6%)** | 42  (80.8%) | 45  (76.3%) | **254**  **(80.9%)** | 161  (85.2%) | 92  (74.8%) | **156**  **(81.3%)** | 115  (83.3%) | 41  (75.9%) | **89**  **(80.2%)** | 39  (88.6) | 50  (74.6%) |
|  |  | **TG** | **23**  **(20.5%)** | 9  (17.3%) | 14  (23.7%) | **54**  **(17.2%)** | 25  (13.2%) | 28  (22.8%) | **34**  **(17.7%)** | 22  (15.9%) | 12  (22.2%) | **18**  **(16.2%)** | 3  (6.8%) | 15  (22.4%) |
|  |  | **GG** | **1**  **(0.9%)** | 1  (1.9%) | 0  (0.0%) | **6**  **(1.9%)** | 3  (1.6%) | 3  (2.4%) | **2**  **(1.0%)** | 1  (0.7%) | 1  (1.9%) | **4**  **(3.6%)** | 2  (4.5%) | 2  (3.0%) |
|  |  |  |  |  |  |  |  |  |  |  |  |  |  |  |
| **IL12B** | **rs6870828** | **AA** | **34**  **(34.3%)** | 11  (25.6%) | 22  (40.0%) | **103**  **(34.7%)** | 61  (33.9%) | 41  (35.7%) | **63**  **(34.2%)** | 43  (32.6%) | 20  (38.5%) | **34**  **(33.0%)** | 13  (31.7%) | 21  (33.9%) |
|  |  | **AG** | **52**  **(52.5%)** | 23  (53.5%) | 29  (52.7%) | **147**  **(49.5%)** | 91  (50.6% | 55  (47.8%) | **86**  **(46.7%)** | 68  (51.5%) | 18  (34.6%) | **58**  **(56.3%)** | 22  (53.7%) | 36  (58.1%) |
|  |  | **GG** | **13**  **(13.1%)** | 9  (20.9%) | 4  (7.3%) | **47**  **(15.8)** | 28  (15.6%) | 19  (16.5%) | **35**  **(19.0%)** | 21  (15.9%) | 14  (26.9%) | **11**  **(10.7%)** | 6  (14.6%) | 5  (8.1%) |
|  |  |  |  |  |  |  |  |  |  |  |  |  |  |  |
| **IL12B** | **rs6859018** | **CC** | **49**  **(45.0%)** | 24  (48.0%) | 25  (42.4%) | **147**  **(47.1%)** | 89  (45.5%) | 60  (49.6%) | **88**  **(45.4%)** | 62  (44.3%) | 26  (48.1%) | **57**  **(52.8%)** | 23  (53.5%) | 34  (52.3%) |
|  |  | **CT** | **48**  **(44.0%)** | 23  (46.0%) | 25  (45.4%) | **127**  **(40.7%)** | 76  (40.2%) | 50  (41.3%) | **79**  **(40.7%)** | 58  (41.4%) | 21  (38.9%) | **43**  **(40.4%)** | 16  (37.2%) | 27  (41.5%) |
|  |  | **TT** | **12**  **(11.2%)** | 3  (6.0%) | 9  (15.2%) | **38**  **(12.2%)** | 27  (14.3%) | 11  (9.1%) | **27**  **(13.9%)** | 20  (14.3%) | 7  (13.0%) | **8**  **(7.4%)** | 4  (9.3%) | 4  (6.2%) |
|  |  |  |  |  |  |  |  |  |  |  |  |  |  |  |
| **IL10** | **rs1800890** | **TT** | **57**  **(52.8%)** | 72  (59.0%) | 25  (42.4%) | **171**  **(55.2%)** | 98  (52.7%) | 31  (64.6%) | **106**  **(55.2%)** | 73  (52.9%) | 33  (61.1%) | **58**  **(53.7%)** | 20  (47.6%) | 38  (57.6%) |
|  |  | **TA** | **45**  **(41.7%)** | 46  (37.7%) | 31  (52.5%) | **125**  **(40.3%)** | 78  (41.9%) | 14  (29.2%) | **80**  **(41.7%)** | 59  (42.8%) | 21  (38.9%) | **42**  **(38.9%)** | 18  (42.9%) | 24  (36.4%) |
|  |  | **AA** | **6**  **(5.6%)** | 4  (3.3%) | 3  (5.1%) | **14**  **(4.5%)** | 10  (5.4%) | 3  (6.3%) | **6**  **(3.1%)** | 6  (4.3%) | 0  (0.0%) | **8**  **(7.4%)** | 4  (9.5%) | 4  (6.1%) |
|  |  |  |  |  |  |  |  |  |  |  |  |  |  |  |
| **IL10** | **rs1800896** | **AA** | **45**  **(42.1%)** | 52  (43.0%) | 23  (40.4%) | **131**  **(42.7%)** | 79  (42.9%) | 21  (42.9%) | **78**  **(41.1%)** | 57  (41.9%) | 21  (38.9%) | **48**  **(44.4%)** | 18  (42.9%) | 30  (45.5%) |
|  |  | **AG** | **45**  **(42.1%)** | 64  (52.9%) | 25  (43.9%) | **151**  **(49.2%)** | 85  (46.2%) | 20  (40.8%) | **97**  **(51.1%)** | 67  (49.3%) | 30  (55.6%) | **50**  **(46.3%)** | 16  (38.1%) | 34  (51.5%) |
|  |  | **GG** | **17**  **(15.9%)** | 5  (4.1%) | 9  (15.8%) | **25**  **(8.1%)** | 20  (10.9%) | 8  (16.3%) | **15**  **(7.9%)** | 12  (8.8%) | 3  (5.6%) | **10**  **(9.3%)** | 8  (19.0%) | 2  (3.0%) |
|  |  |  |  |  |  |  |  |  |  |  |  |  |  |  |
| **IL10** | **rs1800871** | **CC** | **50**  **(41.2%)** | 47  (39.8%) | 25  (43.1%) | **126**  **(41.2%)** | 79  (42.5%) | 25  (50.0%) | **79**  **(41.9%)** | 56  (41.2%) | 23  (43.4%) | **45**  **(42.1%)** | 21  (48.8%) | 24  (37.5%) |
|  |  | **CT** | **41**  **(37.6%)** | 56  (47.5%) | 27  (46.6%) | **144**  **(47.1%)** | 86  (46.2%) | 13  (26.0%) | **91**  **(48.1%)** | 66  (48.5%) | 25  (47.2%) | **47**  **(43.9%)** | 16  (37.2%) | 31  (48.4%) |
|  |  | **TT** | **18**  **(16.5%)** | 15  (12.7%) | 6  (10.3%) | **36**  **(11.8%)** | 21  (11.3%) | 12  (24.0%) | **19**  **(10.1%)** | 14  (10.3%) | 5  (9.4%) | **15**  **(14.0%)** | 6  (14.0%) | 9  (14.1%) |
|  |  |  |  |  |  |  |  |  |  |  |  |  |  |  |
| **IL10** | **rs1518111** | **GG** | **52**  **(48.1%)** | 47  (39.5%) | 28  (47.5%) | **131**  **(42.1%)** | 84  (44.2%) | 24  (50.0%) | **82**  **(42.7%)** | 59  (42.4%) | 23  (43.4%) | **47**  **(43.1%)** | 23  (52.3%) | 24  (36.9%) |
|  |  | **GA** | **42**  **(38.9%)** | 56  (47.1%) | 26  (44.1%) | **147**  **(47.3%)** | 89  (46.8%) | 15  (31.3%) | **95**  **(49.5%)** | 70  (50.4%) | 25  (47.2%) | **46**  **(42.2%)** | 15  (34.1%) | 31  (47.7%) |
|  |  | **AA** | **14**  **(13.0%)** | 16  (13.4%) | 5  (8.5%) | **33**  **(10.6%)** | 17  (8.9%) | 9  (18.8%) | **15**  **(7.8%)** | 10  (7.2%) | 5  (9.4%) | **16**  **(14.7%)** | 9  (13.6%) | 10  (15.4%) |
|  |  |  |  |  |  |  |  |  |  |  |  |  |  |  |
| **IL10** | **rs3024496** | **TT** | **38**  **(35.2%)** | 19  (38.8%) | 18  (31.0%) | **120**  **(38.6%)** | 72  (38.3%) | 48  (39.7%) | **73**  **(38.0%)** | 53  (38.4%) | 20  (37.0%) | **42**  **(38.9%)** | 15  (34.9%) | 27  (41.5%) |
|  |  | **TC** | **53**  **(49.1%)** | 23  (46.9%) | 30  (51.7%) | **164**  **(52.7%)** | 96  (51.1%) | 66  (54.5%) | **103**  **(53.6%)** | 72  (52.2%) | 31  (57.4%) | **55**  **(50.9%)** | 21  (48.8%) | 34  (52.3%) |
|  |  | **CC** | **17**  **(15.7%)** | 7  (14.3%) | 10  (17.2%) | **27**  **(8.7%)** | 20  (10.6%) | 7  (5.8%) | **16**  **(8.3%)** | 13  (9.4%) | 3  (5.6%) | **11**  **(10.2%)** | 7  (16.3%) | 4  (6.2%) |
|  |  |  |  |  |  |  |  |  |  |  |  |  |  |  |
| **IL10** | **rs6673928** | **CC** | **112**  **(97.4%)** | 52  (100%) | 59  (95.2%) | **313**  **(98.7%)** | 187  (97.9%) | 124  (100%) | **192**  **(99.0%)** | 138  (98.6%) | 54  (100%) | **110**  **(98.2%)** | 42  (95.5%) | 68  (100%) |
|  |  | **CA** | **3**  **(2.6%)** | 0 (0.0%) | 3  (4.8%) | **4**  **(1.3%)** | 4 (2.1%) | 0 W0.0%) | **2 (1.0%)** | 2 (1.4%) | 0  (0.0%) | **(1.8%)** | 2 (4.5%) | 0  (0.0%) |
|  |  |  |  |  |  |  |  |  |  |  |  |  |  |  |
| **IFNG** | **rs2069705** | **TT** | **38**  **(34.2%)** | 20  (40.0%) | 17  (28.3%) | **118**  **(38.4%)** | 65  (34.9%) | 52  (43.7%) | **78**  **(40.2%)** | 55  (39.3%) | 23  (42.6%) | **36**  **(34.6%)** | 9  (22.0%) | 27  (42.9%) |
|  |  | **TC** | **58**  **(52.3%)** | 22  (44.0%) | 36  (60.0%) | **145**  **(47.2%)** | 99  (53.2%) | 46  (38.7%) | **88**  **(45.4%)** | 67  (47.9%) | 21  (38.9%) | **53**  **(51.0%)** | 28  (68.3%) | 25  (39.7%) |
|  |  | **CC** | **15**  **(13.5%)** | 8  (16.0%) | 7  (11.7%) | **44**  **(14.3%)** | 22  (11.8%) | 21  (17.6%) | **28**  **(14.4%)** | 18  (12.9%) | 10  (18.5%) | **15**  **(14.4%)** | 4  (9.8%) | 11  (17.5%) |
|  |  |  |  |  |  |  |  |  |  |  |  |  |  |  |
| **IFNG** | **rs1861494** | **AA** | **62**  **(54.9%)** | 30  (57.7%) | 31  (51.7%) | **202**  **(64.7%)** | 122  (64.2%) | 79  (65.8%) | **133**  **(67.5%)** | 100  (70.4%) | 33  (60.0%) | **63**  **(60.0%)** | 19  (45.2%) | 44  (69.8%) |
|  |  | **AG** | **47**  **(41.6%)** | 18  (34.6%) | 29  (48.3%) | **103**  **(33.0%)** | 65  (34.2%) | 37  (30.8%) | **61**  **(31.0%)** | 40  (28.2%) | 21  (38.2%) | **38**  **(36.2%)** | 22  (52.4%) | 16  (25.4%) |
|  |  | **GG** | **4**  **(3.5%)** | 4  (7.7%) | 0  (0.0%) | **7**  **(2.2%)** | 3  (1.6%) | 4  (3.3%) | **3**  **(1.5%)** | 2  (1.4%) | 1  (1.8%) | **63**  **(60.0%)** | 1  (2.4%) | 3  (4.8%) |
|  |  |  |  |  |  |  |  |  |  |  |  |  |  |  |
| **IFNG** | **rs2069718** | **CC** | **27**  **(24.3%)** | 15  (30.6%) | 11  (18.0%) | **85**  **(27.3%)** | 48  (25.5%) | 36  (29.8%) | **56**  **(28.4%)** | 40  (28.2%) | 16  (29.1%) | **26**  **(24.8%)** | 7  (17.1%) | 19  (29.7%) |
|  |  | **CT** | **53**  **(47.7%)** | 16  (32.7%) | 37  (60.7%) | **156**  **(50.2%)** | 99  (52.7%) | 57  (47.1%) | **98**  **(49.7%)** | 72  (50.7%) | 26  (47.3%) | **54**  **(51.4%)** | 24  (58.5%) | 30  (46.9%) |
|  |  | **TT** | **31**  **(27.9%)** | 18  (36.7%) | 13  (21.3%) | **70**  **(22.5%)** | 41  (21.8%) | 28  (23.1%) | **43**  **(21.8%)** | 30  (21.1%) | 13  (23.6%) | **25**  **(23.8%)** | 10  (24.4%) | 15  (23.4%) |
|  |  |  |  |  |  |  |  |  |  |  |  |  |  |  |
| **IFNG** | **rs2069727** | **AA** | **44**  **(40.0%)** | 22  (44.9%) | 22  (36.7%) | **120**  **(39.1%)** | 72  (38.5%) | 47  (36.6%) | **76**  **(38.8%)** | 53  (17.3%) | 23  (41.8%) | **40**  **(38.8%)** | 17  (41.5%) | 23  (37.1%) |
|  |  | **AG** | **52**  **(47.3%)** | 21  (42.9%) | 31  (51.7%) | **135**  **(44.0%)** | 82  (43.9%) | 52  (44.1%) | **85**  **(43.4%)** | 61  (43.3%) | 24  (43.6%) | **47**  **(45.6%)** | 19  (46.3%) | 28  (45.2%) |
|  |  | **GG** | **14**  **(12.7%)** | 6  (12.2%) | 7  (11.7%) | **52**  **(16.9%)** | 33  (17.6%) | 19  (16.1%) | **35**  **(17.9%)** | 27  (19.1%) | 8  (14.5%) | **16**  **(15.5%)** | 5  (12.2%) | 11  (17.7%) |
|  |  |  |  |  |  |  |  |  |  |  |  |  |  |  |
| **IFNG** | **rs3181035** | **GG** | **77**  **(68.8%)** | 37  (72.5%) | 40  (65.6%) | **231**  **(73.8%)** | 142  (74.0%) | 87  (73.1%) | **146**  **(73.7%)** | 106  (73.6%) | 40  (74.1%) | **78**  **(74.3%)** | 32  (76.2%) | 46  (73.0%) |
|  |  | **GA** | **32**  **(28.6%)** | 11  (21.3%) | 21  (34.4%) | **78**  **(24.9%)** | 49  (25.5%) | 29  (24.4%) | **51**  **(25.8%)** | 37  (25.7%) | 14  (25.9%) | **24**  **(22.9%)** | 10  (23.8%) | 14  (22.2%) |
|  |  | **AA** | **3**  **(2.7%)** | 3  (5.9%) | 0  (0.0%) | **4**  **(1.3%)** | 1  (0.5%) | 3  (2.5%) | **1**  **(0.5%)** | 1  (0.7%) | 0  (0.0%) | **3**  **(2.9%)** | 0  (0.0%) | 3  (4.8%) |
|  |  |  |  |  |  |  |  |  |  |  |  |  |  |  |
| **IL4** | **rs2070874** | **CC** | **64**  **(55.7%)** | 28  (53.8%) | 36  (57.1%) | **160 (51.3%)** | 98 (51.6%) | 62  (51.7%) | **96**  **(48.5%)** | 69  (48.3%) | 27  (49.1%) | **59**  **(56.7%)** | 26  (63.4%) | 33  (52.4%) |
|  |  | **CT** | **40**  **(34.8%)** | 20  (38.5%) | 20  (31.7%) | **130 (41.7%)** | 77  (40.5%) | 51  (42.5%) | **86**  **(41.9%)** | 59  (41.3%) | 24  (43.6%) | **42**  **(40.4%)** | 15  (36.6%) | 27  (42.9%) |
|  |  | **TT** | **11**  **(9.6%)** | 4  (7.7%) | 7  (11.1%) | **22**  **(7.1%)** | 15  (7.9%) | 7  (11.1%) | **19**  **(9.6%)** | 15  (10.5%) | 4  (7.3%) | **3**  **(2.9%)** | 0  (0.0%) | 3  (4.8%) |
|  |  |  |  |  |  |  |  |  |  |  |  |  |  |  |
| **IL4** | **rs2227284** | **AA** | **27**  **(24.8%)** | 9  (18.4%) | 18  (30.0%) | **89**  **(29.4%)** | 56  (30.3%) | 33  (28.4%) | **69**  **(35.4%)** | 50  (35.5%) | 19  (35.2%) | **18**  **(18.0%)** | 4  (10.3%) | 14  (23.0%) |
|  |  | **AC** | **48**  **(44.0%)** | 23  (46.9%) | 25  (41.7%) | **136**  **(44.9%)** | 81  (43.8%) | 53  (45.7%) | **83**  **(42.6%)** | 59  (41.8%) | 24  (44.4%) | **48**  **(48.0%)** | 20  (51.3%) | 28  (45.9%) |
|  |  | **CC** | **34**  **(31.2%)** | 17  (34.7%) | 17  (28.3%) | **78**  **(25.7%)** | 48  (25.9%) | 30 (25.9%) | **43**  **(22.1%)** | 32  (22.7%) | 11  (20.4%) | **34**  **(34.0%)** | 15  (38.5%) | 19  (31.1%) |
|  |  |  |  |  |  |  |  |  |  |  |  |  |  |  |
| **IL4** | **rs2243261** | **GG** | **109**  **(92.4%)** | 47  (88.7%) | 61  (95.3%) | **287**  **(91.4%)** | 180  (93.8%) | 105  (87.5%) | **181**  **(91.4%)** | 133  (92.4%) | 48  (88.9%) | **96**  **(90.6%)** | 41  (97.6%) | 55  (85.9%) |
|  |  | **GT** | **9**  **(7.6%)** | 6  (11.3%) | 3  (4.7%) | **25**  **(8.0%)** | 12  (6.3%) | 13  (10.8%) | **15**  **(7.6%)** | 11  (7.6%) | 4  (7.4%) | **10**  **(9.4%)** | 1  (2.4%) | 9  (14.1%) |
|  |  | **TT** | **0**  **(0.0%)** | 0  (0.0%) | 0  (0.0%) | **2**  **(0.6%)** | 0  (0.0%) | 2  (1.7%) | **2**  **(1.0%)** | 0  (0.0%) | 2  (3.7%) | **0**  **(0.0%)** | 0  (0.0%) | 0  (0.0%) |
|  |  |  |  |  |  |  |  |  |  |  |  |  |  |  |
| **IL4** | **rs2243268** | **AA** | **73**  **(66.4%)** | 33  (68.8%) | 40  (64.5%) | **188**  **(61.4%)** | 112  (59.6%) | 76  (65.5%) | **112**  **(57.4%)** | 79  (56.0%) | 33  (61.1%) | **69**  **(68.3%)** | 28  (68.3%) | 41  (68.3%) |
|  |  | **AC** | **30**  **(27.3%)** | 13  (27.1%) | 17  (27.4%) | **106**  **(34.6%)** | 69  (36.7%) | 35  (30.2%) | **73**  **(37.4%)** | 55  (39.0%) | 18  (33.3%) | **30**  **(29.7%)** | 13  (31.7%) | 17  (28.3%) |
|  |  | **CC** | **7**  **(6.4%)** | 2  (4.2%) | 5  (8.1%) | **12**  **(3.9%)** | 7  (3.7%) | 5  (8.1%) | **10**  **(5.1%)** | 7  (5.0%) | 3  (5.6%) | **2**  **(2.0%)** | 0  (0.0%) | 2  (3.3%) |
|  |  |  |  |  |  |  |  |  |  |  |  |  |  |  |
| **IL4** | **rs2243274** | **GG** | **54**  **(49.5%)** | 25  (52.1%) | 29  (47.5%) | **131**  **(42.7%)** | 80  (42.8%) | 51  (43.2%) | **76**  **(39.2%)** | 53  (37.9%) | 23  (42.6%) | **51**  **(49.0%)** | 23  (56.1%) | 28  (44.4%) |
|  |  | **GA** | **42**  **(38.5%)** | 20  (41.7%) | 22  (36.1%) | **132**  **(43.0%)** | 81  (43.3%) | 49  (41.5%) | **87**  **(44.8%)** | 65  (46.4%) | 22  (40.7%) | **40**  **(38.5%)** | 14  (34.1%) | 26  (41.3%) |
|  |  | **AA** | **13**  **(11.9%)** | 3  (6.3%) | 10  (16.4%) | **44**  **(14.3%)** | 26  (13.9%) | 18  (15.3%) | **31**  **(16.0%)** | 22  (15.7%) | 9  (16.7%) | **13**  **(12.5%)** | 4  (9.8%) | 9  (14.3%) |
|  |  |  |  |  |  |  |  |  |  |  |  |  |  |  |
| **IL4** | **rs2243290** | **CC** | **71**  **(62.3%)** | 31  (63.3%) | 40  (62.5%) | **174**  **(56.5%)** | 105  (56.1%) | 69  (58.0%) | **105**  **(53.8%)** | 74  (52.5%) | 31  (57.4%) | **63**  **(61.2%)** | 27  (67.5%) | 36  (57.1%) |
|  |  | **CA** | **32**  **(28.1%)** | 15  (30.6%) | 17  (26.6%) | **115**  **(37.3%)** | 69  (36.9%) | 44  (37.0%) | **74**  **(37.9%)** | 54  (38.3%) | 20  (37.0%) | **37**  **(35.9%)** | 13  (32.5%) | 24  (38.1%) |
|  |  | **AA** | **11**  **(9.6%)** | 3  (6.1%) | 7  (10.9%) | **19**  **(6.2%)** | 13  (7.0%) | 6  (5.0%) | **16**  **(8.2%)** | 13  (9.2%) | 3  (5.6%) | **3**  **(2.9%)** | 0  (0.0%) | 3  (4.8%) |
|  |  |  |  |  |  |  |  |  |  |  |  |  |  |  |
| **IL4** | **rs2406539** | **AA** | **96**  **(85.0%)** | 41  (80.4%) | 54  (88.5%) | **244**  **(81.9%)** | 146  (80.7%) | 96  (83.5%) | **153**  **(80.5%)** | 110  (79.7%) | 43  (82.7%) | **85**  **(84.2%)** | 34  (85.0%) | 51  (83.6%) |
|  |  | **AT** | **17**  **(15.0%)** | 10  (19.6%) | 7  (11.5%) | **53**  **(17.8%)** | 35  (19.3%) | 18 (15.7%) | **36**  **(18.9%)** | 28  (20.3%) | 8  (15.4%) | **16**  **(15.8%)** | 6  (15.0%) | 10  (16.4%) |
|  |  | **TT** | **0**  **(0.0%)** | 0  (0.0%) | 0  (0.0%) | **1**  **(0.3%)** | 0  (0.0%) | 1  (0.9%) | **1**  **(0.5%)** | 0  (0.0%) | 1  (1.9%) | **0**  **(0.0%)** | 0  (0.0%) | 0  (0.0%) |
|  |  |  |  |  |  |  |  |  |  |  |  |  |  |  |
